# Transporter-dependent uptake and metabolism of myocardial interstitial serotonin in the rat heart

**DOI:** 10.1186/s12576-022-00852-2

**Published:** 2022-10-26

**Authors:** Takashi Sonobe, Tsuyoshi Akiyama, James T. Pearson

**Affiliations:** 1 Department of Cardiac Physiology, National Cerebral and Cardiovascular Center Research Institute, Suita, Osaka Japan; 2 Department of Physiology, Victoria Heart Institute and Monash Biomedicine Discovery Institute, Monash University, Melbourne, Australia

**Keywords:** Cardiac microdialysis, Serotonin transporter, Plasma membrane monoamine transporter, Monoamine oxidase, 5-HT, 5-HIAA

## Abstract

To investigate the roles of the serotonin (5-HT) transporter (SERT) and plasma membrane monoamine transporter (PMAT) in 5-HT uptake and its metabolism in the heart, we monitored myocardial interstitial levels of 5-HT and 5-HIAA, a metabolite of 5-HT by monoamine oxidase (MAO), in anesthetized rats using a microdialysis technique. Fluoxetine (SERT inhibitor), decynium-22 (PMAT inhibitor), or their mixture was locally administered by reverse-microdialysis for 60 min. Subsequently, pargyline (MAO inhibitor) was co-administered. Fluoxetine rapidly increased dialysate 5-HT concentration, while decynium-22 gradually increased it. The mixture induced a larger increase in dialysate 5-HT concentration compared to fluoxetine or decynium-22 alone. Fluoxetine increased dialysate 5-HIAA concentration, and this increase was abolished by pargyline. Decynium-22 and the mixture did not change dialysate 5-HIAA concentration, which were not affected by pargyline. Both SERT and PMAT regulate myocardial interstitial 5-HT levels by its uptake; however, 5-HT uptake via PMAT leads to 5-HT metabolism by MAO.

## Background

Myocardial interstitial serotonin (5-hydroxytryptamine, 5-HT) plays important roles in the heart through various subtypes of 5-HT receptor [1]. 5-HT induces vascular smooth muscle contraction via 5-HT 1B and 2A receptors [2, 3]. 5-HT affects cardiac contractility via 5-HT 2B receptors [4] and induces positive inotropic and chronotropic effects via 5-HT 4A receptors (in human heart atrium) [5]. Moreover, 5-HT activates afferent cardiac vagal nerves via 5-HT3 receptors [6, 7] and inhibits the release of norepinephrine from cardiac sympathetic nerve terminals via 5-HT1B/1D receptors [8]. Myocardial interstitial 5-HT is partly washed out into the blood stream and partly taken up into cells of cardiac tissue via 5-HT transporters. Intracellular 5-HT is subjected to deamination by monoamine oxidase (MAO) and subsequently degraded to 5-hydroxyindole acetic acid (5-HIAA) [9]. Thus, 5-HT uptake via a transporter and degradation by MAO may play an important role in the regulation of cardiac functions [10–13]. In the heart, serotonin transporter (SERT) exists on the membrane of platelets in the blood stream [14] and plasma membrane monoamine transporter (PMAT) exists on the plasma membrane of cardiomyocytes [5, 15]; however, the roles of the two transporters in 5-HT uptake and subsequent 5-HT metabolism remain unclear in the heart in vivo.

In this study, to investigate the contribution of SERT and PMAT to 5-HT uptake and subsequent metabolism, we applied a microdialysis technique to the heart of anesthetized rats. We simultaneously monitored myocardial interstitial levels of 5-HT and 5-HIAA and examined the effect of inhibitors of SERT, PMAT, and MAO.

## Methods

### Ethical approval

Animal experiments were conducted in accordance with the Guide for the Care and Use of Laboratory Animals, 8th edition (the National Academies Press) and approved by the Institutional Animal Care and Utilization Committee of the National Cerebral and Cardiovascular Center Research Institute (No. 20045, 21060). Reporting of animal experiments in this study also complies with the ARRIVE guidelines for animal research [16].

**Surgical preparation** A total of 14 male Wistar rats (Japan SLC), weighing 493 ± 36 g (~ 18–28 weeks) were used. We only used male rats to avoid periodic hormonal changes associated with the estrous cycle in female rats from confounding the experimental results. Rats were anaesthetized by pentobarbital sodium (60 mg/kg i.p.) supplemented with an analgesic agent, butorphanol (0.25 mg/kg i.p.). The rats were then quickly intubated and mechanically ventilated (3 ml, 80 breath/min) with room air mixed with oxygen. A PE-50 catheter was cannulated to the jugular vein for maintaining the anesthesia by continuous infusion of pentobarbital (20 mg/kg/h) and butorphanol (0.1 mg/kg/h). A similar catheter was inserted into the carotid artery for measuring arterial blood pressure. With the rats in a lateral position, a left thoracotomy was performed, and a dialysis probe was implanted transversely into the lateral wall of the left ventricle [17–20]. Two probes were implanted at least 5 mm apart from each other. The implanted probe was found to be broken at the end of the experiment on a few occasions due to movement of the heart, in which case all dialysate samples from the broken probe were discarded. Materials and properties of the microdialysis probe have been described previously [21]. At the end of the experiment, the rats were sacrificed with an overdose of pentobarbital sodium (150 mg/kg i.v. bolus), and then a post-mortem examination was conducted to confirm that the dialysis probe did not penetrate into the ventricular cavity.

### Measurement

Arterial blood pressure was measured via a pressure transducer (BD DTXPlus, BD). The pressure signal was relayed to a BP Amp (ML117, AD Instruments) and digitized using a data acquisition system (Power Lab 16/35, AD Instruments). Heart rate (HR) was determined by peak detection of the pressure signal on LabChart7 software (AD Instruments). The body temperature was monitored with a rectal thermistor and maintained at around 37 °C using a heating pad and a lamp throughout the experiment.

### Cardiac microdialysis technique

**Fig. 1:**
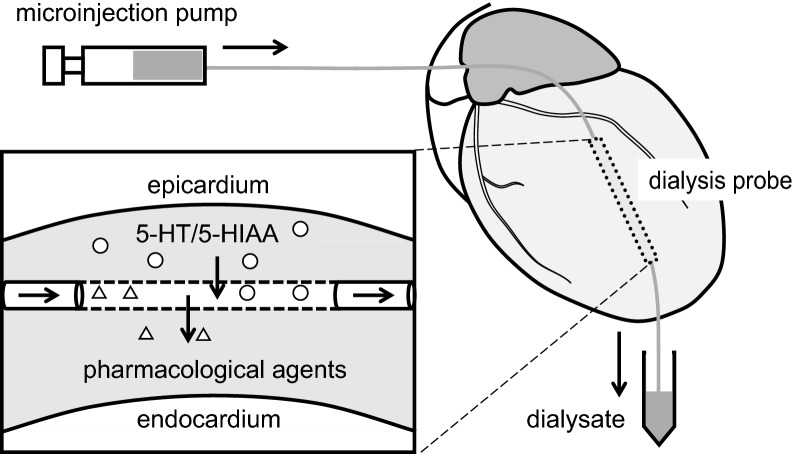
Schematic illustration of the microdialysis technique in the heart in vivo. A dialysis probe is implanted transversely into the lateral wall of the left ventricle. Myocardial interstitial 5-HT and 5-HIAA diffuses into perfusate across the dialysis membrane. Pharmacological agents diffuse into the interstitium according to a concentration gradient by reverse-microdialysis

Figure 1 shows a scheme of the cardiac microdialysis and reverse-microdialysis technique. The implanted microdialysis probe was continuously perfused with Ringer’s solution or Ringer’s solution containing pharmacological agents at an infusion rate of 2 µl/min using a microinjection pump (CMA/100, Carnegie Medicine). Because diffusion across the semipermeable membrane is required, based on previous results [19, 20, 22, 23], we used the pharmacological agent at concentrations 10–100 times higher than that required in experimental settings in vitro. To minimize an acute effect of surgical invasion due to the probe implantation, dialysate sampling was started from at least 2 h after the initiation of perfusion. Duration for each dialysate sampling was 15 min, which yielded a volume of 30 µl. Concentrations of 5-HT and 5-HIAA in the dialysate samples were measured by high performance liquid chromatography with electro-chemical detection (HPLC-ECD 700 series, Eicom) as previously described [18, 20, 24]. In our previous study [20], we have confirmed that measured dialysate concentration of 5-HT and 5-HIAA are stable and did not significantly change at least during 60 min of sampling period in the absence of pharmacological agents.

### Experimental protocols

**Fig. 2:**
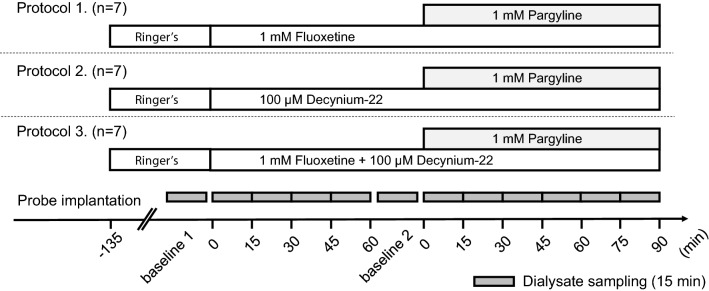
Experimental protocols. In protocol 1 (*n* = 7), after baseline dialysate sampling (baseline 1), perfusate (Ringer's solution) was switched to 1 mM fluoxetine. The drug administration was continued for 60 min, then second baseline dialysate sampling was performed (baseline 2). The perfusate was then switched to 1 mM fluoxetine mixed with 1 mM pargyline. The drug administration was continued for 90 min. In protocol 2 (*n* = 7) and 3 (*n* = 7), similar experiments as protocol 1 were performed in the presence of 100 µM decynium-22 and the mixture of fluoxetine and decynium-22, respectively

A summary of the experimental protocols is shown in Fig. 2. After baseline dialysate sampling (baseline 1), the perfusate (Ringer’s solution) was switched to (1) 1 mM fluoxetine, (2) 100 µM decynium-22, or (3) mixture of 1 mM fluoxetine and 100 µM decynium-22. Fluoxetine (Fujifilm Wako Pure Chemical) was used to investigate the contribution of SERT to uptake of myocardial interstitial 5-HT and decynium-22 (Tocris Bioscience) was used to investigate the contribution of PMAT to the uptake of myocardial interstitial 5-HT. The drug administration was continued for 60 min, and thus 4 consecutive samples were collected. After the new baseline dialysate sampling (baseline 2), 1 mM pargyline, a monoamine oxidase inhibitor, was co-administered with either fluoxetine, decynium-22, or the mixture of fluoxetine and decynium-22 for 90 min, and 6 consecutive samples were collected to investigate 5-HT metabolism by MAO following to 5-HT uptake into cells. In our previous studies using the same experimental setup, we found that local administration of 1 mM fluoxetine by reverse microdialysis increased 5-HT level at ~ 5 times higher than the baseline and this level was maintained after 60 min from initiation of the perfusion [20]. Similarly, we found that 100 µM decynium-22 significantly increased 5-HT level and became stable after 60 min from initiation of the perfusion [18]. We also confirmed that local administration of 1 mM fluoxetine or100 µM decynium-22 by reverse-microdialysis had no systemic effect. Therefore, 1 mM fluoxetine or 100 µM decynium-22 administration was chosen in this study and the administration was started at 60 min before the co-administration with pargyline. The effect of 1 mM pargyline alone on the dialysate 5-HT concentration was also tested and found significant [22].

### Statistical analysis

All statistical analyses were conducted with GraphPad Prism 8 (GraphPad Software, San Diego, CA, USA). All results were presented as means ± SD. Differences among the 3 groups were compared by one-way ANOVA followed by Tukey’s multiple comparison test. Time course changes in 5-HT and 5-HIAA were compared by one-way repeated measures ANOVA followed by Sidak’s multiple comparison tests to find a significant difference from baseline 1 (*P < 0.05) and from baseline 2 (†*P* < 0.05).

## Results

### Time course of heart rate and mean arterial blood pressure

HR and mean arterial blood pressure (MABP) were stable throughout the experiment (Table 1). Although there were some time-dependent changes in both HR and MABP, there were no significant differences among the groups.

**Table 1:** Time course of heart rate and mean arterial pressure

HR (bpm)	Baseline 1	Ringer 7.5 min	Ringer 22.5 min	Ringer 37.5 min	Ringer 52.5 min	Baseline 2	Parg 7.5 min	Parg 22.5 min	Parg 37.5 min	Parg 52.5 min	Parg 67.5 min	Parg 82.5 min
Fluo + Parg	342 ± 40	349 ± 36	352 ± 35	353 ± 35	352 ± 33	352 ± 33	351 ± 31	349 ± 31	348 ± 31	349 ± 30	350 ± 31	350 ± 31
D22 + Parg	344 ± 26	351 ± 24	355 ± 20	354 ± 22	353 ± 23	351 ± 24	348 ± 22	346 ± 20	346 ± 19	347 ± 18	349 ± 18	349 ± 19
Fluo + D22 + Parg	341 ± 25	348 ± 25	352 ± 23 *	354 ± 22 *	354 ± 22 *	353 ± 22 *	350 ± 21	348 ± 20	347 ± 21	346 ± 23	347 ± 24	347 ± 25

MABP (mmHg)	Baseline 1	Ringer 7.5 min	Ringer 22.5 min	Ringer 37.5 min	Ringer 52.5 min	Baseline 2	Parg 7.5 min	Parg 22.5 min	Parg 37.5 min	Parg 52.5 min	Parg 67.5 min	Parg 82.5 min
Fluo + Parg	95 ± 27	96 ± 24	97 ± 22	95 ± 19	94 ± 18	94 ± 20	92 ± 17	91 ± 16	91 ± 16	91 ± 15	92 ± 15	92 ± 14
D22 + Parg	99 ± 18	99 ± 17	99 ± 15	96 ± 16	94 ± 16	92 ± 16	90 ± 16*	89 ± 14*	89 ± 14*	89 ± 14*	90 ± 14*	90 ± 13*
Fluo + D22 + Parg	99 ± 23	99 ± 19	98 ± 18	97 ± 15	96 ± 15	94 ± 14	91 ± 13	90 ± 13	88 ± 13*	88 ± 14*	89 ± 15	89 ± 14

Values are shown in mean ± SD (*n* = 7). Effects of local administration of fluoxetine (Fluo), decynium-22 (D22), and Fluo + D22 co-administered with pargyline (Parg) on heart rate (HR) and mean arterial blood pressure (MABP) were compared by one-way repeated measures ANOVA followed by Tukey's multiple comparison tests

^*^p < 0.05 vs baseline 1

### Dialysate 5-HT and 5-HIAA concentration at baseline 1 (Figs. 3, 4, and 5)

**Fig. 3:**
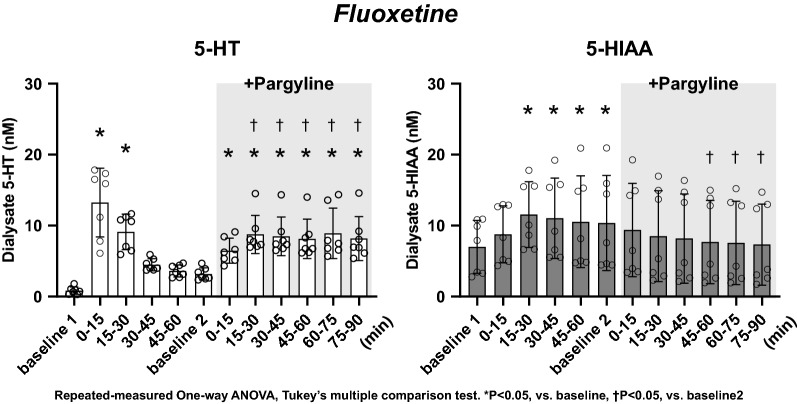
Effect of local administration of 1 mM fluoxetine and following co-administration of 1 mM pargyline on dialysate concentrations of 5-HT and 5-HIAA. The data are shown as mean ± SD with individual data points (*n* = 7). *p < 0.05 vs baseline 1. †*p* < 0.05 vs baseline 2. Repeated-measures one-way ANOVA, Tukey’s multiple comparison test

**Fig. 4:**
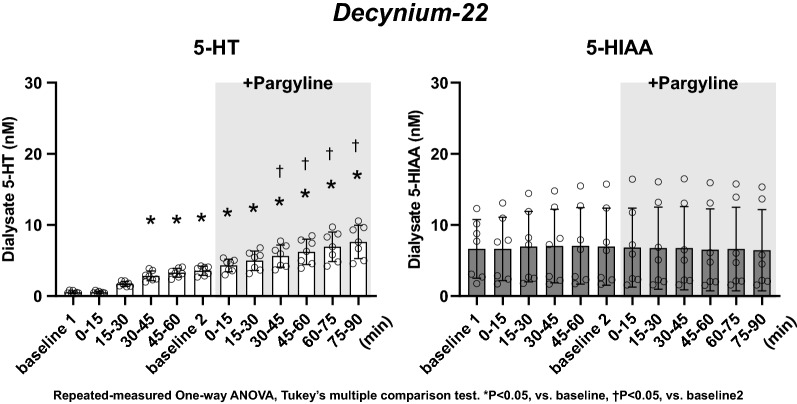
Effect of local administration of 100 µM decynium-22 and following co-administration of 1 mM pargyline on dialysate concentrations of 5-HT and 5-HIAA. The data are shown as mean ± SD with individual data points (*n* = 7). *p < 0.05 vs baseline 1. †*p* < 0.05 vs baseline 2. Repeated-measures one-way ANOVA, Tukey’s multiple comparison test

**Fig. 5:**
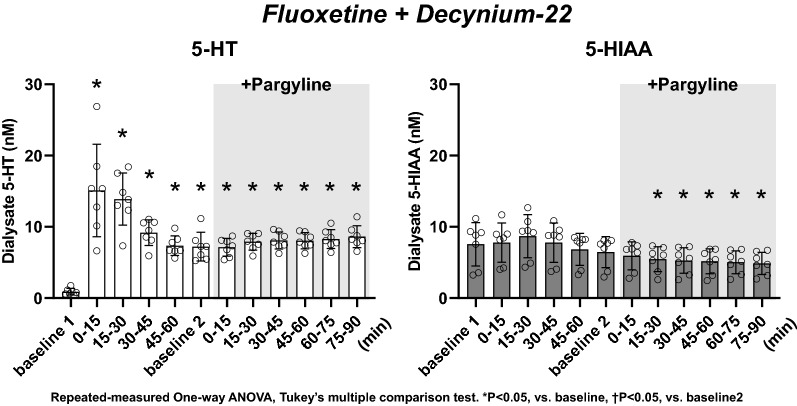
Effect of local administration of the mixture of 1 mM fluoxetine and 100 µM decynium-22 and following co-administration of 1 mM pargyline on dialysate concentrations of 5-HT and 5-HIAA. The data are shown as mean ± SD with individual data points (*n* = 7). *p < 0.05 vs baseline 1. Repeated-measures one-way ANOVA, Tukey’s multiple comparison test

Dialysate 5-HT concentration at baseline 1 was 0.85 ± 0.51, 0.55 ± 0.19, and 0.89 ± 0.46 nM in the groups treated with fluoxetine, decynium-22, and fluoxetine + decynium-22, respectively. Dialysate 5-HIAA concentration at baseline 1 was 7.02 ± 3.73, 6.64 ± 4.13, and 7.55 ± 3.03 nM in the groups treated with fluoxetine, decynium-22, and fluoxetine + decynium-22, respectively. There were no significant differences in dialysate 5-HT and 5-HIAA concentration at baseline 1 among the groups.

### Dialysate 5-HT and 5-HIAA concentration in the presence of fluoxetine (Fig. 3)

Fluoxetine increased dialysate 5-HT concentration to about 16 times of baseline 1 at 0–15 min (13.24 ± 4.84 nM, *P < 0.05 vs baseline (1). The dialysate 5-HT concentration then declined and became stable at 45–60 min of administration (3.61 ± 0.84 nM). Fluoxetine also increased dialysate 5-HIAA concentration at 15–30 min (11.56 ± 4.63 nM, *P < 0.05 vs baseline 1), and dialysate 5-HIAA concentration did not return to the level of baseline 1 during the 60-min sampling period.

In the presence of fluoxetine, pargyline further increased dialysate 5-HT concentration (8.75 ± 2.69 nM at 15–30 min, †*P* < 0.05 vs baseline (2). Dialysate 5-HT concentration remained at this high level during the 90-min sampling period. Pargyline decreased dialysate 5-HIAA concentration from baseline 2 level (7.33 ± 5.71 nM at 75–90 min, †*P* < 0.05 vs baseline 2).

### Dialysate 5-HT and 5-HIAA concentration in the presence of decynium-22 (Fig. 4)

Decynium-22 gradually increased dialysate 5-HT concentration. The dialysate 5-HT concentration reached 3.28 ± 0.63 nM at 45–60 min of administration (*P < 0.05 vs baseline 1). Meanwhile, decynium-22 did not change dialysate 5-HIAA concentration during the 60-min sampling period.

In the presence of decynium-22, pargyline further increased dialysate 5-HT concentration (7.62 ± 2.33 nM at 75–90 min, †*P* < 0.05 vs baseline 2), but did not change dialysate 5-HIAA concentration.

### Dialysate 5-HT and 5-HIAA concentration in the presence of fluoxetine and decynium-22 (Fig. 5)

Combined administration of fluoxetine and decynium-22 increased dialysate 5-HT concentration similar to fluoxetine alone (15.10 ± 6.49 nM at 0–15 min, *P < 0.05 vs baseline 1), except that dialysate 5-HT concentration at 45–60 min was significantly higher than baseline 1 (*P < 0.05). Combined administration of fluoxetine and decynium-22 did not change dialysate 5-HIAA concentration during the 60-min sampling period.

In the presence of both fluoxetine and decynium-22, pargyline did not further increase dialysate 5-HT concentration but maintained dialysate 5-HT concentration at a higher level during the 90-min sampling period compared to baseline 1 (*P < 0.05). Pargyline did not change dialysate 5-HIAA concentration, but dialysate 5-HIAA concentration decreased to a level below baseline 1 (4.86 ± 1.56 nM at 75–90 min, *P < 0.05).

## Discussion

Fluoxetine (SERT inhibitor) and decynium-22 (PMAT inhibitor) increased myocardial interstitial 5-HT level with different time-course effects, and the response of myocardial interstitial 5-HT to the mixture of fluoxetine and decynium-22 was almost, the calculated sum of the individual responses to fluoxetine and decynium-22. Fluoxetine increased myocardial interstitial 5-HIAA level, but decynium-22 and the mixture of fluoxetine and decynium-22 did not change myocardial interstitial 5-HIAA level. Additional administration of pargyline decreased myocardial interstitial 5-HIAA level from baseline 2 in the presence of fluoxetine but not in the presence of decynium-22 and mixture of fluoxetine and decynium-22. These findings suggest that both SERT and PMAT independently contribute to maintain a low level of myocardial interstitial 5-HT; however, 5-HT uptake via PMAT leads to 5-HT metabolism by MAO, while 5-HT uptake via SERT does not.

### Regulation of myocardial interstitial 5-HT level by 5-HT transporters

It is important to consider that SERT is mainly expressed on the membrane of platelets and is expressed less on the membrane of cardiomyocytes [5]. The initial rapid increase in myocardial interstitial 5-HT level by fluoxetine indicated that SERT of platelets potently regulates myocardial interstitial 5-HT level. However, myocardial interstitial 5-HT level declined after 15 min of fluoxetine administration regardless of continuous administration of fluoxetine. This decline of myocardial interstitial 5-HT level might be due to the activity of alternate 5-HT transporters, which are insensitive to fluoxetine. When the myocardial interstitial 5-HT level started to increase above the normal/physiological range, the role of high-capacity transporters (e.g., PMAT) could become predominant by 2–3 orders of magnitude higher maximal capacity to transport than those of SERT [25]. In other words, the contribution of PMAT to 5-HT uptake into the cells increased to prevent further increase in the myocardial interstitial 5-HT level. Changes in SERT translocation on the cellular membrane might also affect the dynamic change in interstitial 5-HT concentration, since the distribution of SERT is modulated according to 5-HT concentration between the outside and inside of the membrane [26].

In contrast to the rapid increase by fluoxetine, decynium-22 increased myocardial interstitial 5-HT level more slowly. Inhibition of decynium-22-sensitive 5-HT transporter increased the interstitial 5-HT level, but the transporter-dependent rate of the increase in 5-HT could be limited due to the low-affinity of PMAT for 5-HT [27, 28]. In addition, decynium-22-insensitve 5-HT transporter, e.g., SERT has been reported to indicate 2–3 order magnitude high affinity for 5-HT [25]. This property also might prevent the increase in interstitial 5-HT level in the presence of decynium-22.

High-affinity SERT-mediated 5-HT uptake is functional at low 5-HT level [29]. In this low-5-HT condition, the 5-HT that is taken up into platelets might be stored in the vesicles and reused, avoiding unnecessary loss of 5-HT. Meanwhile, high-capacity PMAT-mediated 5-HT uptake becomes increasingly prominent at high 5-HT levels, i.e., in the presence of excessive amount of 5-HT. In that case, 5-HT uptake through PMAT followed by the 5-HT degradation by MAO would play an important role in the clearance of 5-HT.

### *Metabolism by MAO after uptake *via* 5-HT transporters*

Fluoxetine increased myocardial interstitial 5-HIAA level and additional pargyline reduced myocardial interstitial 5-HIAA level. Moreover, decynium-22 and mixture of fluoxetine and decynium-22 did not change myocardial interstitial 5-HIAA level in spite of the increase in myocardial interstitial 5-HT level and additional pargyline did not change myocardial interstitial 5-HIAA level from baseline 2. These results indicate that, in the presence of fluoxetine, myocardial interstitial 5-HT was taken up via the intact decynium-22-sensitive transporter and metabolized to 5-HIAA by MAO, and that, in the presence of decinium-22, myocardial interstitial 5-HT was taken up via intact SERT but not metabolized to 5-HIAA by MAO. Thus, we conclude that 5-HT uptake via PMAT leads to 5-HT metabolism by MAO, but 5-HT uptake via SERT does not.

MAO is distributed widely in various cells including platelets, sympathetic nerves, and cardiomyocytes in the heart and SERT is mainly expressed on the membrane of platelet. At present we cannot explain the reason why 5-HT uptake via SERT does not lead to 5-HT metabolism by MAO. Platelets might concentrate 5-HT to the vesicle and reuse 5-HT. Although the cells contributing to 5-HT degradation cannot be specified, 5-HT transporters contributing 5-HT degradation in the heart is specified.

5-HT taken up via PMAT is metabolized by MAO and maintains the 5-HT gradient between cytosol and extra-cellular space by decreasing intracellular 5-HT level. On the other hand, 5-HT taken up via SERT does not get metabolized by MAO. It has been reported that SERT is a high-affinity but low-capacity transporter for 5-HT but PMAT is a low-affinity, high-capacity transporter for 5-HT. 5-HT degradation by MAO could partly explain the difference in properties of the two transporters.

### *Role of degradation by MAO in uptake *via* 5-HT transporters*

In the presence of fluoxetine, pargyline increased myocardial interstitial 5-HT level. Meanwhile, in the presence of both fluoxetine and decynium-22, myocardial interstitial 5-HT level was already high before pargyline and additional pargyline did not change myocardial interstitial 5-HT level any further. These results suggest that administration of pargyline induces the inhibition of 5-HT uptake via decyniumu-22 sensitive transporter. Inhibition of MAO increases intracellular 5-HT level by reduction of 5-HT degradation. This increased intracellular 5-HT could prevent 5-HT uptake via PMAT due to the decrease in 5-HT concentration gradient between intra- and extracellular space. Thus, we consider that intracellular 5-HT degradation by MAO maintains 5-HT uptake via PMAT.

In the presence of decynium-22, however, myocardial interstitial 5-HT level continued to increase after pargyline. Continuous increase in myocardial interstitial 5-HT after pargyline in the presence of decynium-22 might be due to continuous effect of decynium-22 but not due to the effect of pargyline.

### Limitation

Although we have observed that time-course changes in dialysate 5-HT and 5-HIAA are different between fluoxetine and decynium-22, we cannot exclude the possible interference due to the route of drug administration. The reverse-microdialysis technique that was used to administer the pharmacological agents in this study requires relatively high concentration to cross the dialysis membrane and to diffuse into the interstitium according to a concentration gradient. This diffusion capacity in the myocardial interstitium could be different between fluoxetine and decynium-22, suggesting that if one crosses the membrane and reaches interstitium surrounding the dialysis probe much faster than the other, the time-course changes in 5-HT and 5-HIAA mig**h**t be dependent on the diffusion capacity of the pharmacological agents.

It might be helpful to confirm SERT or PMAT dependent 5-HT uptake in mice using transgenic models instead of using pharmacological agents. However, genetically engineered mice occasionally show altered non-specific expression of compensatory genes/proteins, and present complicated compensatory effects through activation of other mechanisms. For example, organic cation transporter (OCT), which has an affinity for 5-HT was upregulated in SERT knockout mice [30, 31]. This effect may hinder our ability to understand the kinetics of 5-HT/5-HIAA in the heart in vivo that we found in this study. This point may be a potential option for future study after careful consideration of an appropriate model.

### Summary

**Fig. 6:**
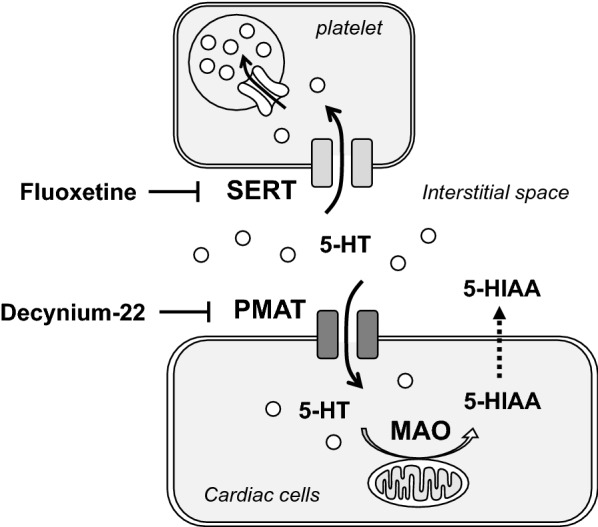
Putative mechanisms regulating cardiac interstitial level of 5-HT in a physiological condition. Interstitial level of 5-HT in the heart is maintained low mostly by fluoxetine-sensitive transporter (SERT) dependent uptake into platelets and stored in intracellular vesicles. This stored 5-HT in the vesicle could be reused and less metabolized. Meanwhile, interstitial 5-HT is also taken up by decynium-22 sensitive transporter (PMAT) into cardiac cells. The transported 5-HT is then metabolized by MAO to produce 5-HIAA

Putative mechanisms regulating myocardial interstitial 5-HT level under physiological condition are summarized in Fig. 6. Myocardial interstitial 5-HT is taken up into platelets by fluoxetine-sensitive, high-affinity transporter (SERT) to maintain low level of the myocardial interstitial 5-HT, but is less metabolized and could be reused. Meanwhile, myocardial interstitial 5-HT is also taken up into cardiac cells by decynium-22-sensitive, high-capacity transporter (PMAT), which is then metabolized by MAO to produce 5-HIAA.

## Conclusions

Both SERT and PMAT independently regulate myocardial interstitial 5-HT levels by its uptake. 5-HT uptake via PMAT rather than SERT contributes more to intracellular 5-HT metabolism by MAO.

## Data Availability

The data supporting the results in this study are available from the corresponding author upon reasonable request.
